# Editorial: Neuromodulation in Basic, Translational and Clinical Research in Psychiatry

**DOI:** 10.3389/fnhum.2019.00438

**Published:** 2019-12-20

**Authors:** Ryouhei Ishii, Keiichiro Nishida, Nagy A. Youssef, Kay Jann, Shun Takahashi

**Affiliations:** ^1^Graduate School of Comprehensive Rehabilitation, Osaka Prefecture University, Habikino, Japan; ^2^Department of Psychiatry, Osaka University Graduate School of Medicine, Suita, Japan; ^3^Neuropsychiatry, Kansai Medical University, Moriguchi, Japan; ^4^Medical College of Georgia, Augusta University, Augusta, GA, United States; ^5^USC Stevens Neuroimaging and Informatics Institute, Keck School of Medicine of USC, University of Southern California, Los Angeles, CA, United States; ^6^Department of Neuropsychiatry, Wakayama Medical University, Wakayama, Japan

**Keywords:** neuromodulation, electroconvulsive therapy (ECT), repetitive transcranialmagnetic stimulation (rTMS), transcranial direct current stimulation (tDCS), neurofeedback, psychiatry, rehabilitation, resting state network (RSN)

There has been emerging evidence of non-pharmacologic therapeutics for psychiatric illnesses to modulate brain activity. These methodologies are often referred as “neuromodulation,” which is a broad term that could technically be considered to cover any medical, surgical, or physiologic therapy designed to alter the function of the nervous system in some manner. In the clinical neurosciences, however, neuromodulation is understood to refer specifically to therapies that involve targeted delivery of electrical current or magnetic field, which includes electroconvulsive therapy (ECT), one of the oldest treatments in psychiatry, and vagus nerve stimulation (VNS), approved by the Food and Drug Administration (FDA) in 2005 for severe depression, and repetitive transcranial magnetic stimulation (rTMS), approved by the FDA in 2008 for the treatment of major depression. Recently, studies using transcranial direct current stimulation (tDCS), electric trigeminal nerve stimulation (eTNS), deep brain stimulation (DBS), and neurofeedback have been also reported in a growing trend. To develop more effective treatments for psychiatric diseases, translational approaches bridging basic and clinical evidence deserve considerations.

In this e-book, we tried to provide a forum for researchers interested in basic, translational, and clinical research of neuromodulation for psychiatric illnesses and aim to facilitate an integrative view of neuromodulation. It was our unexpected pleasure to have 16 papers in this topic which reported new exciting findings and cutting edge methodologies. The included papers were divided into four groups as follows: (1) the clinical application of tDCS, ECT, and rTMS for psychiatric diseases, (2) the brain function enhancement by tDCS, (3) new application of neurofeedback, and (4) translational research in neuromodulation.

The clinical application of neuromodulation for psychiatric diseases, mainly mood disorders and cognitive impairments, was described in the papers of the first group. To examine the effects of ECT on neuronal oscillatory pattern and phase synchronization, and the relationship between clinical response or cognitive change and electroencephalogram (EEG) measurements, Takayima et al. analyzed resting 19-lead EEG data recorded from 13 depressed patients before and after a course of ECT by exact low resolution electromagnetic tomography (eLORETA). They found ECT modulation on resting-state EEG oscillatory patterns and phase synchronization in central nodes of the default mode network (DMN) and suggested that changes in beta synchronization in the left hemisphere might explain the ECT-related cognitive side effects. Nishida et al. evaluated the immediate impact on anxiety of tDCS to the left dorsolateral prefrontal cortex (DLPFC) or dorsomedial prefrontal cortex (DMPFC) in 14 patients with major depressive disorder (MDD) and 19 healthy controls (HCs) and its association with pre-stimulus brain activity by using eLORETA. They suggested that the association between pre-tDCS brain activity and the anxiety reduction effect of tDCS depends on psychopathology (depressed or non-depressed) as well as the site of stimulation (DMPFC or left DLPFC) and insomnia. To compare rTMS-induced cortical plasticity changes in patients with MDD and in healthy volunteers, Vignaud et al. used motor evoked potentials (MEPs) evoked by single-pulse TMS before and after a single and continuous intermittent theta-burst stimulation (TBS). They observed impaired TBS-induced neuroplasticity in patients with MDD compared to that in controls and suggested impaired long-term potentiation (LTP)-like mechanisms in MDD. Kazemi et al. reported the effects of bilateral rTMS of DLPFC on the activity of resting state network (RSN) as well as relevant cognitive function in patients with bipolar depression that responded to treatment. They suggested that bilateral rTMS of DLPFC changed the activity of RSN and consequently improves verbal memory and executive functions in patients with bipolar depression. Inagawa et al. assessed the safety and efficacy of tDCS during cognitive training on cognitive functioning in patients with mild or major neurocognitive disorders by adopting two-arm, parallel, randomized, and sham-controlled trial and reported tDCS is safe and tolerable but causes no statistically significant cognitive effects in patients with mild or major neurocognitive disorders. Cruz Gonzalez et al. conducted the systematic review on the literature about the efficacy of tDCS in improving cognitive outcomes in mild cognitive impairment (MCI) and dementia, including 12 studies with 195 patients with dementia and four studies with 53 patients with MCI. They concluded that tDCS improves memory in dementia in the short term and have a mild positive effect on memory and language in MCI. These studies showed clinical validity and usefulness of neuromodurational methodology with the effectiveness on brain activity and pathophysiology related to certain brain areas and connectivity related to various types of cognitive process and psychiatric symptoms.

The second group of papers investigated the possibility of the enhancement of brain function of normal subjects by applying tDCS. To test whether anodal offline tDCS over the left prefrontal cortex (PFC) enhances working memory (WM) capacity by modulating the oscillatory activity in the left dorsolateral PFC (DLPFC) using magnetoencephalography (MEG), Ikeda et al. investigated the cortical oscillatory changes induced by anodal tDCS during a WM task. They elucidated no-correlation between stable WM capacity and increased gamma-band oscillation induced by tDCS. Gold and Ciorciari applied tDCS to “flow states,” considered a positive, subjective experience during an optimal balance between skills and task demands. Although they found the increased flow experience by real stimulation for both trained and untrained Tetris players compared to sham stimulation, improved performance effects were only seen with untrained groups. They concluded that tDCS may encourage flow experiences in complex real-life motor tasks that occur during sports, games, and everyday life. Steinberg et al. reviewed the literature about acute behavioral, neurophysiological, and neurochemical effects and the mechanistic pathways of tDCS and aerobic exercise (AE) and discusses potential interactions and synergies between tDCS and AE that might be provoked when directly combining both techniques. They suggested that a direct combination of tDCS and AE provides multiple beneficial opportunities for synergistic effects both within non-clinical settings in health and for treating several psychiatric and neurologic conditions. These papers proved quite wide and promising utility of tDCS on the brain function enhancement and augmentation which would provide huge markets for normal subjects.

The third group contains the new application of neurofeedback from Karch et al. assessing the combination of real-time fMRI (rtfMRI) and neurofeedback (NF) to predict the outcome of NF training plus group psychotherapy at the beginning of the treatment for patients with tobacco use disorder. They reported that they could estimate a successful withdrawal in patients with tobacco use disorder by analyzing the first rtfMRI NF session: a pronounced reduction of frontal responses during NF training in patients might be the functional correlate of better therapeutic success. They suggested that the results of the first NF sessions could be useful as predictor whether a patient will be able to achieve success after the behavioral group therapy and NF training in quitting smoking or not. Chiba et al. conducted a systematic review to compare Decoded Neurofeedback (DecNef) effect with those of conventional EEG/fMRI-based neurofeedback on post-traumatic stress disorder (PTSD) amelioration. They suggested that DecNef could be a promising therapy that bypasses the unpleasantness of conscious exposure associated with conventional therapies for fear related disorders, including PTSD. The other types of neurofeedback studies, especially electrophysiological procedures, which have some advantages like cheaper running costs, smaller apparatus, and non-invasiveness without any exposure to radiation or strong magnetic fields, would be encouraged and provoked by these MRI neurofeedback studies.

The fourth group of articles tried to expand the new frontiers for translational research of neuromodulation, combining electroacupuncture and neurogenesis in PTSD rats (Zhou et al.), TMS and brain-derived neurotrophic factor (BDNF) in fibromyalgia and depression (Cardinal et al.), acoustic startle response (ASR), and locomotor dynamics in autism spectrum disorder (ASD) (Ebishima et al.; Ogino et al.), P300 and heart rate in emotional processing (Matsuo et al.). The future translational approach bridging between clinical application on neuropsychiatric diseases and basic pathophysiological research about the mechanism of these neuromodurational methodologies will be expected.

In summary, this e-book presented novel methodologies and various applications of neuromodulation in psychiatry. As a result, these papers established the feasibility and plausibility of neuromodulation in psychiatry with new evidence and threw impacts on new directions for expanding new possibility of neuromodulation for basic and clinical application.

## Author Contributions

RI and KN discussed about this Research Topic and wrote the manuscript. NY, KJ, and ST gave significant advice and helped the editing the manuscript.

### Conflict of Interest

NY discloses that he receives research support (but not salary support) from Merck & Co., VistaGen Therapeutics, Inc., and MECTA Corporation, the U.S. Department of Veterans Affairs, and August Biomedical Research Corporation. The remaining authors declare that the research was conducted in the absence of any commercial or financial relationships that could be construed as a potential conflict of interest.

